# Pathogenic *Escherichia coli* producing Extended-Spectrum β-Lactamases isolated from surface water and wastewater

**DOI:** 10.1038/srep14372

**Published:** 2015-09-24

**Authors:** Eelco Franz, Christiaan Veenman, Angela H. A. M. van Hoek, Ana de Roda Husman, Hetty Blaak

**Affiliations:** 1 National Institute for Public Health and the Environment (RIVM), Centre for Infectious Disease Control, Bilthoven, The Netherlands

## Abstract

To assess public health risks from environmental exposure to Extended-Spectrum β-Lactamases (ESBL)-producing bacteria, it is necessary to have insight in the proportion of relative harmless commensal variants and potentially pathogenic ones (which may directly cause disease). In the current study, 170 ESBL-producing *E. coli* from Dutch wastewater (n = 82) and surface water (n = 88) were characterized with respect to ESBL-genotype, phylogenetic group, resistance phenotype and virulence markers associated with enteroaggregative *E. coli* (EAEC), enteroinvasive *E. coli* (EIEC), enteropathogenic *E. coli* (EPEC), enterotoxigenic *E. coli* (ETEC), extraintesinal *E. coli* (ExPEC), and Shiga toxin-producing *E. coli* (STEC). Overall, 17.1% of all ESBL-producing *E. coli* were suspected pathogenic variants. Suspected ExPECs constituted 8.8% of all ESBL-producing variants and 8.3% were potential gastrointestinal pathogens (4.1% EAEC, 1.8% EPEC, 1.2% EIEC, 1.2% ETEC, no STEC). Suspected pathogens were significantly associated with ESBL-genotype CTX-M-15 (X^2^ = 14.7, P < 0.001) and phylogenetic group B2 (X^2^ = 23.5, P < 0.001). Finally, 84% of the pathogenic ESBL-producing *E. coli* isolates were resistant to three or more different classes of antibiotics. In conclusion, this study demonstrates that the aquatic environment is a potential reservoir of *E. coli* variants that combine ESBL-genes, a high level of multi-drug resistance and virulence factors, and therewith pose a health risk to humans upon exposure.

*Escherichia coli* is generally considered a commensal inhabitant of the gastrointestinal tract of humans and animals. Consequently, it is one of the most frequently used indicator bacterium for fecal contamination in environments. However, *E. coli* is also one of the most important causes of nosocomial-acquired and community-acquired infections in humans and can easily gain resistance to antibiotics consumed by humans and animals^1^. This includes resistance caused by extended-spectrum β-lactamases (ESBLs). Like other antibiotic resistances, genes for ESBLs are most often encoded on plasmids, which can readily be transferred between bacteria^2^. In the Netherlands, ESBL-producing *E. coli* strains are present among the commensal *E. coli* population in healthy individuals and food-producing animals^3^,^4^ and have been isolated from various surface waters^5^. Surface water is an important source for drinking water production and is used for recreation and irrigation of crops, providing exposure of humans to ESBL-*E. coli*^6^. Exposure to ESBL-producing pathogenic *E. coli* variants may directly result in hard-to-treat infection, also in healthy individuals.

*E. coli* is characterized by a rather clonal population structure in which isolates form four main phylogenetic groups (A, B1, B2, D)^7^. Pathogenic *E. coli* are scattered throughout the phylogenetic tree of the species but phylogenetic groups vary in their host association, presence of virulence factors and persistence in the non-host environment^8^,^9^,^10^. Strains isolated from non-host environments are generally more likely to belong to phylogenetic group B1 and A^9^,^10^. Strains of phylogenetic group B1 seem to be more host generalists whereas B2 strains being preferentially host-adapted and are more likely to harbor traits for extraintestinal infection^8^. Pathogenic *E. coli* can roughly be separated into two groups: those with primarily gastrointestinal disease and the ones causing extraintestinal infections. The intestinal pathogens can be further distinguished in diffusely adherent *E. coli* (DAEC), enteroaggregative *E. coli* (EAEC), enterohemorrhagic *E. coli* (EHEC; which in turn is a subgroup of Shiga toxin-producing *E. coli* or STEC), enteroinvasive *E. coli* (EIEC), enteropathogenic *E. coli* (EPEC), enterotoxigenic *E. coli* (ETEC), and extraintestinal *E. coli* (ExPEC) . ExPEC includes avian pathogenic *E. coli* (APEC), neonatal meningitis *E. coli* (NMEC) and uropathogenic *E. coli* (UPEC)^11^. The differences in the ability of *E. coli* strains to cause disease and the diverse syndromes caused by the various pathotypes can be attributed to specific virulence genes and the variability in which these occur among strains^11^.

Few studies have investigated the pathogenic potential of environmental ESBL-producing bacteria which may be directly linked to public health risk^12^. The presence of virulence factors, representative for different pathogenic groups of *E. coli*, among ESBL-*E. coli* isolated from wastewater and surface water was examined in the current study. In addition, the distribution of phylogenetic groups was determined in order to examine correlations between ESBL-production, virulence factors and genetic background. This sheds light on the transmission ecology and direct public health risks of exposure to waterborne ESBL-*E. coli.*

## Materials and Methods

### Isolates

ESBL-producing isolates were selected from an existing collection of isolates obtained from wastewater and surface water sampled between 2010 and 2012^5^,^13^. Surface water samples (n = 20) were taken at ten different sites situated in four different regions in the Netherlands (including rivers, canals, lakes, North Sea), wastewater samples (n = 20) included influents and effluents from wastewater treatment plants (WWTP), an international airport WWTP, and from wastewater of health care institutions. Isolates were obtained by filtration of multiple volumes of water samples through 0.45 μm filters, followed by incubation of these filters on selective culture media for the isolation of ESB-producing *E. coli*: Tryptone Bile X-glucuronide medium supplemented with 1 μg/ml cefotaxime (TBX/CTX) or on ChromID^TM^ ESBL agar (Biomerieux). Incubation conditions were 18–24 hours at 36 ± 2 °C or 4 to 5 hours at 36 ± 2 °C followed by 18 to 19 hours at 44 ± 0.5 °C. Isolation procedures were based on standard isolation procedures for the selective isolation of *E. coli* from water and food using chromogenic media (NEN-EN-ISO 9308-1 and ISO 16649-2), adapted to enable selective growth of ESBL-producing variants. Variations in isolation procedures was related to isolates being obtained as part of different projects. Suspected ESBL-*E. coli* isolates (i.e. the ß-glucuronidase-positive colonies on TBX-CTX as well as on ChromID^TM^ ESBL agar) were further confirmed as *E. coli* by testing for indole-production using BBL Dry Slide^TM^ (BD), and subsequently tested for ESBL-production by disk diffusion following CLSI guidelines^14^, using Sensi-Discs^TM^ (BD, Breda, the Netherlands). Zone diameters were determined for cefotaxime (30 μg) ± clavulanic acid (10 μg), ceftazidime (30 μg) ± clavulanic acid (10 μg). ESBL-producing isolates were defined as strains resistant to cefotaxime (zone diameter ≤22 mm) and/or ceftazidime (zone diameter ≤17 mm), and an increase in zone diameter of ≥5 mm with the disks containing clavulanic acid^14^.

From this pre-existing collection of confirmed ESBL-producing *E. coli* isolates, 93 wastewater and 93 surface water isolates, from 20 samples each, were selected more or less randomly, but taking into account established ABR profiles (when available), to minimize the chance of including duplicate isolates from the same sample. After characterization of phylogenetic groups, ESBL-genes, virulence genes and antibiotic resistance profiles was completed, some isolates were retrospectively identified as duplicates and omitted, leaving 88 wastewater (63 from WWTP, 15 from the international airport and 10 from health care institutions) and 82 surface water isolates for analyses.

### Molecular characterization

Each isolate was characterized with respect to phylogenetic group, ESBL-genotype, and the presence of virulence factors. For this purpose, material from one single colony was suspended in Tris EDTA buffer (pH 8.0, Sigma-Aldrich, Zwijndrecht, the Netherlands), and DNA extract stored at −20 °C. PCRs were carried out using primers and conditions as described in Supplementary Table S1.

For phylogenetic group analysis, a multiplex PCR targeting *chuA*, *yjaA* genes and TspE4.C2 DNA fragment as described by Clermont *et al.*^15^ was used. Strains were sub-grouped according to Escobar-Páramo *et al.*^16^: subgroup A_0_, *chuA*−, *yjaA*−, TspE4.C2−; subgroup A_1_, *chuA*−, *yjaA*+, TspE4.C2−; group B1, *chuA*−, *yjaA−*, TspE4.C2+; subgroup B2_2_, *chuA*+, *yjaA*+, TspE4.C2−; subgroup B2_3_, *chuA*+, *yjaA*+, TspE4.C2+; subgroup D_1_, *chuA*+, *yjaA*−, TspE4.C2−; subgroup D_2_, *chuA*+, *yjaA*− , TspE4.C2+. For ESBL-genotyping, the presence of *bla*_CTX-M-1_-group, *bla*_CTX-M-2_-group, and *bla*_CTX-M-9_-group, *bla*_OXA_, *bla*_SHV_ and *bla*_TEM_, was established by multiplex PCRs using primers described by Dalenne *et al.*^17^. Products of the expected size were treated with ExoSAP-IT (GE Healthcare, Hoevelaken, the Netherlands) and sequenced using the same primers used to generate PCR-products, and BigDye Terminator v3.1 Cycle Sequencing kit (Applied Biosystems, Bleiswijk, the Netherlands). Thus obtained partial sequences were compared with ESBL-gene sequences in the GenBank database and on the Lahey website (www.lahey.org/Studies). Partial sequence analysis allows identification of clusters of homologous alleles: CTX-M-1/61, CTX-M-3/22/66, CTX-M-9/51, CTX-M-14/21/83/90/113, CTX-M-15/28, CTX-M-27/98, CTX-M-32, CTX-M-55/57, SHV-12/129, TEM-52/92. For sake of annotation, the lowest allele number belonging to a cluster was assigned to identified genotypes. For analysis of virulence factors, PCRs were used targeting genes encoding the diarrheagenic virulence factors: Shigatoxin 1 and 2 (*stx1, stx2*) for STEC, intimin (*eae*) for EPEC, aggregative virulence regulator (*aggR*) for EAEC, invasion plasmid antigen (*ipaH*) for EIEC and heat labile enterotoxin (*estA*) and heat stabile enterotoxine (*eltB*) for ETEC^18^. The following markers were selected for identifying extraintestinal pathogenic *E. coli* (ExPEC): afimbrial adhesion (*afa*), F1C fimbriae (*focG*), cytolytic protein toxin (*hlyD*), iron acquisistion system (*iutA*), group 2 polysaccharide capsule (*kpsMII*), P fimbriae (*papA*) and S fimbriae (*sfaS*)^5^,^13^. ExPEC was defined as strains having three or more of these genes^20^.

### Biofilm formation

Isolates were tested for their capacity to form biofilms using the procedure described by Wakimoto *et al.*^21^. Biofilm formation was defined as having an OD of at least four times that of negative control wells (i.e. moderate and strong biofilm formation according to Naves at al. 2008^22^).

### Analysis of antibiotic resistance

Isolates were screened for antibiotic susceptibility to a panel of 14 antibiotics of human and veterinary clinical relevance, using Sensi-disks^TM^ according to the manufacturers’ instructions (Becton, Dickinson BV, Breda, the Netherlands). Included antibiotics were one or two representatives from eight classes: beta-lactams: ampicillin (penicillins), cefotaxime and ceftazidime (3^rd^ generation cephalosporins); β-lactam/β-lactamase combination: amoxicillin/clavulanic acid; carbapenems: imipenem and meropenem; tetracyclines: tetracycline; (fluor)quinolones: ciprofloxacin and naladixic acid; aminoglycosides: streptomycin and gentamycin; folate pathway inhibitors: sulfisoxazole and trimethoprim; phenicols: chloramphenicol. Resistance was defined as having an inhibition zone smaller than or equal to the clinical break-points defined by CLSI^14^. Multi-drug resistance was defined as resistance to 3 or more different classes of antibiotics^23^.

### Statistics

Differences in frequencies of genetic genotypes and pathogenicity markers among groups was evaluated using chi-squared tests (χ^2^) on contingency tables with a significance level of *P* = 0.05. Univariate analysis of variance was performed for inference on differences in average numbers of pathogenicity markers and multi-drug resistance between ESBL-genotypes and phylogenetic groups. Analyses were performed in IBM SPSS Statistics version 19.

## Results

### Distribution of phylogenetic groups among ESBL-producing E. coli from water

**Figure 1:**
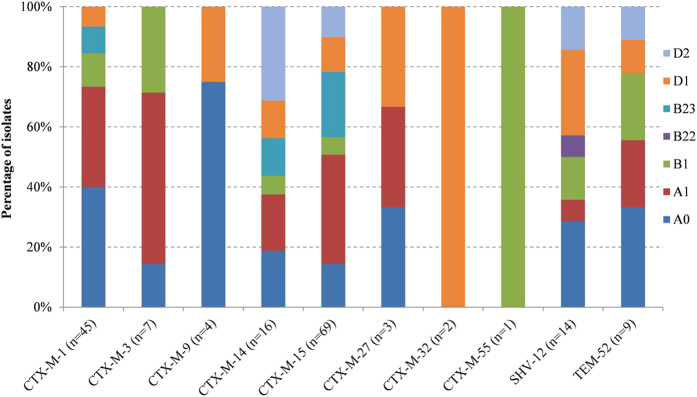
Distribution of phylogenetic subgroups among ESBL-genotypes. Different colors represent the percentages of isolates belonging to the different phylogenetic subgroups for each ESBL-genotype. At the x-axis, the total number of isolates per ESBL-genotype is indicated between brackets.

Among the ESBL-producing *E. coli* from both surface water and wastewater, ten different genotypes were represented. The most prevalent ESBL-genotypes in both types of water were CTX-M-15 (41%) and CTX-M-1 (26%), which together constituted 65% and 74% of all ESBL-producing *E. coli* in surface water and wastewater respectively. Other observed genotypes were CTX-M-14 (9.4%), SHV-12 (8.2%), TEM-52 (5.3%), CTX-M-3 (4.1%), CTX-M-9 (2.4%), CTX-M-27 (1.8%), CTX-M-32 (1.2%) and CTX-M-55 (0.6%). With the exception of the two least prevalent genotypes which were solely detected in wastewater, all were detected in both surface water and wastewater. With respect to phylogenetic groups, the majority of isolates were of group A (55%: A_0_ 25%, A_1_, 30%), followed by group D (22%: D_1_ 13%, D_2_ 9%), B2 (13%: B2_2_ 0.6%, B2_3_ 12%), and B1 (10%). Among wastewater isolates, all phylogenetic (sub)groups were represented, while in the surface water isolates B1, B2_2_ and D_2_ were not detected. In order to determine the distribution of ESBL-genotypes among phylogenetic groups, all isolates were pooled for analysis. With the exception of the two least prevalent ESBL-genotypes (CTX-M-32 and CTX-M-57), all ESBL-genes were detected in multiple *E. coli* backgrounds (Fig. 1). Phylogenetic groups generally associated with commensal *E. coli* (A and B1) as well as the pathogenic D group, had representatives among eight of the ten different ESBL-genotypes. By contrast, *E. coli* of phylogenetic (sub)groups B2 and D_2_ were each observed among four different ESBL-genotypes. Significant associations were identified between ESBL-genotype CTX-M-1 and phylogenetic groups A and D, CTX-M-15 and B2, and SHV-12 and D (Table 1).

### Distribution of virulence markers among ESBL-producing E. coli isolates from water

Overall, 17.1% (29/170) of all ESBL-producing *E. coli* isolates from surface water and wastewater assessed in this study were potentially pathogenic, as defined by the presence of characteristic virulence markers (Table 2). Potential pathogens were significantly associated with ESBL-genotype genotype CTX-M-15 (21/29 among potential pathogens versus 48/141 among non-pathogens) (X^2^ 14.7; P < 0.001; OR = 5.1). In addition, potential pathogens were significantly associated with phylogenetic group B2 (X^2^ = 23.5, P < 0.001, OR = 8.8).

**Figure 2:**
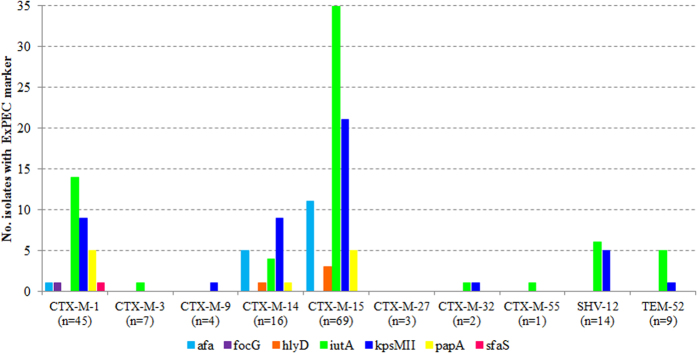
Distribution of ExPEC markers among ESBL-producing *E. coli* isolates. Indicated are the numbers of isolates carrying the indicated ExPEC markers, for each ESBL-genotype. At the x-axis, the total number of isolates per ESBL-genotype are indicated between brackets.

Suspected ExPECs (defined as isolates with three or more ExPEC markers) constituted 8.8% (15/170) of all ESBL-producing isolates: 6.1% of the surface water isolates and 11.4% of the wastewater isolates (Table 2). No significant associations were identified between ExPEC and ESBL-genotype. However, ExPEC was significantly associated with phylogenetic group B2 (X^2^ 42.2, P < 0.001, OR = 23.8). Ninety-eight ESBL-producing isolates (58%) harbored at least one ExPEC marker with *iutA* (39%) and *kpsMII* (28%) the most frequently observed and detected in eight and seven of all ESBL-genotypes, respectively (Fig. 2). The markers *afa* (10%), *papA* (6.5%), and *hlyD* (2.4%) were only observed associated with CTX-M-1-, CTX-M-14- and/or CTX-M-15-genotypes; *focG* and *sfaS* were each observed only once (0.6% of isolates), in relation with CTX-M-1. Significant positive associations were observed between *kpsMIII* and ESBL-genotype CTX-M-14 and CTX-M-27, between *iutA* and CTX-M-15, and between *afa* and, CTX-M-15 and CTX-M-14; a negative association was observed between afa and CTX-M-1 (Table 1). In addition, significant associations were identified between *kpsMIII* and phylogenetic group A (negative), B2 (positive) and D (positive); between *iutA* and phylogenetic group B2 (positive); between *hlyD* and phylogenetic group A (negative) and B1 (positive); and between *afa* and phylogentic group A (negative) (Table 1).

Suspected diarrheagenic variants constituted 8.3% of all ESBL-producing *E. coli* isolates from water (8.5% and 7.8% from surface water and wastewater, respectively) (Table 2). The most prevalent pathogenic variants were EAEC (4.1% of all isolates), EPEC (1.8%), EIEC (1.2%) and ETEC (1.2%). STEC were not detected among the 170 ESBL-producing *E. coli* analyzed. Only three *aggR* positive isolates showed strong biofilm formation (all three OD_strain_/OD_blanco_ = 12). These isolates belonged to phylogenetic group and ESBL genotype: A0/CTX-M-15, A1/CTX-M-15, A1/CTX-M-27. The other four *aggR* positive isolates did not distinguish themselves from the other *E. coli* with respect to biofilm formation (average OD_strain_/OD_blanco_ = 1.2). Potential diarrheagenic *E. coli* was significantly associated with ESBL-genotype CTX-M-15 (X^2^ = 8.3, P = 0.004, OR 2.8). No associations were found with phylogenetic group.

### Multidrug-resistance

Overall, 77% of the ESBL-producing *E. coli* isolates were resistant to three or more different classes of antibiotics (i.e. were multidrug-resistant (MDR)). The proportion MDR *E. coli* was slightly (but not statistically different) higher in suspected pathogenic variants compared to isolates not identified as pathogenic (84% vs. 75%, p > 0.1 Chi-square Test). Two of three suspected EAEC variants (both with strong biofilm forming capacity), all seven isolates carrying EIEC, EPEC, or ETEC markers, and 12 of 15 (80%) of suspected ExPEC variants were multidrug-resistant.

**Figure 3:**
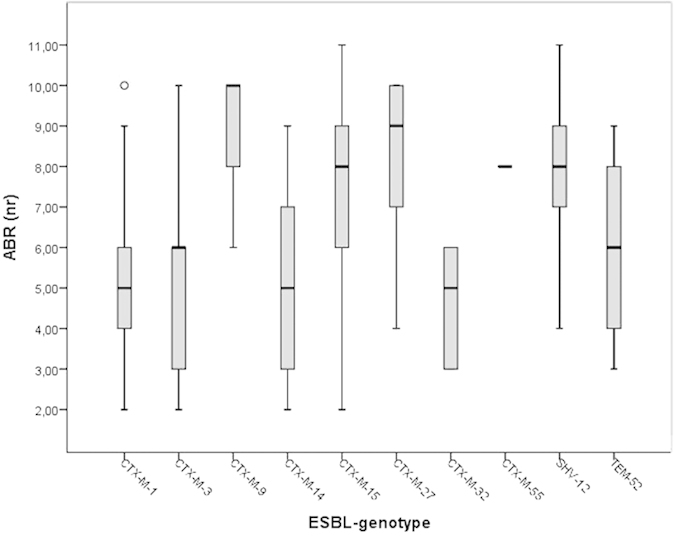
Boxplot of number of antimicrobial resistances (ABR) among isolates of with different ESBL-genotypes. Solid horizontal line represents the median, the box displays the 25%–75% quartile range, the stems show the minimum and maximum values, the circle indicate an outlier.

Suspected pathogenic variants carried on average 6.8 different resistance types (Table 3). No differences was observed between ExPEC and non-ExPEC (P = 0.814), nor between the different phylogenetic groups (P = 0.843). The various ESBL-genotypes differed in the number antibiotics the isolates were resistant to (P < 0.001), with highest level of multi-drug resistance among CTX-M-9, CTX-M-15, CTX-M-27 and SHV-12 (Fig. 3).

## Discussion

Besides health care settings and food, the environment is likely to have a role in the dissemination of ESBL-producing bacteria and may serve as an exposure route to humans. Previously recreational waters were identified as a potential exposure source of ESBL-producing *E.* coli^5^. Even though (outside the clinical setting) *E. coli* is generally considered a relatively harmless inhabitant of the human (and animal) gut, major public health risks may be associated with the spread of ESBL-producing commensal bacteria. Firstly, upon colonization ESBL-producing commensal bacteria may disseminate and transfer ESBL-encoding genes to intestinal pathogens through horizontal gene transfer^24^. Secondly, while relatively harmless for healthy individuals, these opportunistic bacteria may cause disease in more vulnerable individuals, such as hospitalized individuals, the elderly or newborns. Thirdly, exposure to ESBL-producing pathogenic *E. coli* variants may directly result in hard-to-treat infection, also in healthy individuals. The public health impact of exposure to ESBL-producing *E. coli* (and other AMR commensal bacteria) is determined by the sum of these individual risks.

The current study showed that a considerable fraction of ESBL-producing *E. coli* from Dutch surface water and wastewater are potential diarrheagenic or extraintestinal pathogenic. Thus far, only few studies have examined the pathogenicity of non-clinical ESBL-producing *E. coli* strains. A recent study from South Korea reported 60% of ESBL-producing *E. coli* isolated from a river to be potentially pathogenic, which is markedly higher than our findings^12^. These difference in fractions of pathogenic variants between studies possibly reflects differences in water management or differences in antimicrobial selection pressure within humans since extraintestinal pathogenic *E. coli* primarily have a human reservoir^11^. Previously, we have shown concentrations of ESBL-producing *E. coli* ranging from 1.5 to 150 colony forming units (cfu)/liter in recreational waters^5^. Quantitative risk assessment based on these concentrations established an average probability of exposure to ESBL-producing *E. coli* of 0.20 per swimming event for children, and slightly lower for men (0.16) and women (0.13) (Schijven *et al.* unpublished). Combined with the results of the present study the probability of exposure to a pathogenic ESBL-producing *E. coli* becomes 0.03 (0.20 × 0.15) which, given frequent recreation in surface waters, can rise to a considerable cumulative probability of exposure. Recreational freshwater swimming has been identified as a significant risk factor for acquiring urinary tract infection caused by ESBL-producing enterobacteriaceae^6^. The results of the present study provides experimental evidence to this epidemiological association. Ingestion of surface water may lead to intestinal colonization of extraintestinal ESBL-*E. coli* and subsequent urinary tract infection.

The predominance of CTX-M-15 among ESBL-producing *E. coli* in Dutch water and its strong association with virulence factors is in agreement with CTX-M-15 being one of the most common ESBL-genotypes in humans and often being health-care related (i.e. community onset urinary tract infections or nosocomial infections), in the Netherlands and globally^25^,^26^,^27^. The strong association with virulence factors is also consistent with the observed association between CTX-M-15 and phylogenetic group B2 in the present as well as a previous study^27^, which is thought to be more virulent than other groups^28^,^29^. The global occurrence of CTX-M-15-producing ESBL-*E. coli* is partially associated with the spread of the pathogenic ST131 clone, causing urinary tract and bloodstream infections worldwide^30^. Two of the 15 B2_3_/CTX-M-15 isolates included in the current study were characterized with respect to sequence type, and were also identified as ST131 (data not shown). The second prevalent ESBL-genotype among the Dutch water isolates was CTX-M-1. Although globally this genotype was not identified as a major human genotype^26^, in the Netherlands it has previously been recognized as highly prevalent, among humans (the second most prevalent genotype after CTX-M-15)^25^ as well as broilers^31^. The results from this study showed that the microbiological contents of surface- and wastewater provide a reflection of what is circulating among the human population.

This study did not detect any Shiga toxin-producing *E. coli* (STEC) which might be due to several reasons. ESBL-positive STEC seems to be a rare phenomenon^35^. In addition the isolation method used did not select for the isolation of the most frequent STEC serotype O157 since this type, in contrast to other *E. coli*, is β-glucuronidase-negative. It remains unknown why STEC still represent a minor subpopulation of strains that have acquired ESBL genes. A possible explanation might be the relatively low selection pressure in the bovine reservoir (due to lower use of antibiotics^4^), as well as in humans since clinical STEC infections are rarely treated with antibiotics. However, there may be a risk of STEC emerging in the aquatic environment, since Stx-phages are widely distributed and can be acquired by *E. coli* belonging to various phylogenetic and pathogenic groups, including EAEC (for example the German Stx-producing EAEC O104:H4 outbreak)^32^,^33^.

Biofilm formation may be an important contributory factor in persistent infections by allowing the bacteria to evade the local immune system and by preventing the transport of antibacterial factors, including antibiotics. Assays to quantify biofilm formation have been suggested as a possible method of screening for pathogenic EAEC strains^34^. In the current study strong biofilm formation was observed with three out of seven *aggR* positive isolates, which raises the question whether the remaining four isolates can truly be considered potential EAEC based on the detection of *aggR* only. However, extensive studies have been performed using PCR tests to find the right combination of genes identifying the “truly pathogenic” EAEC strains but no consensus has been reached on this matter^35^. The *aggR* gene is highly conserved among EAEC strains and has been found to be associated with diarrhea in several studies^35^. We conclude that all *aggR* positive strains identified in this study can be considered at least as potential EAEC. In addition to the diarrheagenic nature of EAEC these strains also have been associated with urinary tract infections (UTIs) made possible by a combination of EAEC (*aggR*) and ExpEC virulence factors (among which *iutA*)^36^. Three of the *aggR* positive isolates in the current study were simultaneously positive for the ExPEC marker *kpsMIII* and another 3 isolates positive for *iutA*, making these isolates potential hybrid EAEC-ExPEC strains.

In conclusion, this study has shown that environmental ESBL-producing *E. coli* not only are an indirect public health threat by being harmless commensal carriers of ESBL-genes, but also poses a direct threat upon exposure by actually carrying virulence factors representative for major pathogenic groups of *E. coli.* Surface waters and wastewater are a potential reservoir of *E. coli* combining ESBL-genes, high level of multi-drug resistance, and virulence factors. These findings demonstrate the role of the environment in the transmission ecology of pathogenic, and in particular extraintestinal pathogenic, ESBL-producing *E. coli.*

## Additional Information

**How to cite this article**: Franz, E. *et al.* Pathogenic *Escherichia coli* producing Extended-Spectrum β-Lactamases isolated from surface water and wastewater. *Sci. Rep.* **5**, 14372; doi: 10.1038/srep14372 (2015).

## Supplementary Material

Supplementary info Table S1

## Figures and Tables

**Table 1:** Significant (χ^2^ P < 0.05) positive and negative associations between ESBL-genotype, virulence markers and phylogenetic groups.

Marker/group^1^	ESBL-genotype					Phylogenetic group			
	CTX-M-1	SHV-12	CTX-M-15	CTX-M-14	CTX-M-27	A	B1	B2	D
*kpsMIII*	ns	ns	ns	χ^2^ 7.2; OR 3.9 (1.4–11)	χ^2^ 6.6; OR 13.4 (1.1–169)	χ^2^ 47.5; OR 0.06 (0.02–0.15)	ns	χ^2^ 43.6; OR 27.1 (7.5–98.1)	χ^2^ 16.5 OR 4.6 (2.1–10.0)
*iutA*	ns	ns	χ^2^ 6.2; OR 2.2 (1.2–4.2)	ns	ns	ns	ns	χ^2^ 6.2; OR 3.1 (7.5–98.1)	ns
*hlyD*	ns	ns	ns	ns	ns	χ^2^ 5.1; OR 0.95 (0.90–1.00)	χ^2^ 7.3; OR 10.1 (1.3–76.7)	ns	ns
*afa*	χ^2^ 4.1; OR 0.2 (0.1–1.2)	ns	χ^2^ 4.6; OR 3.0 (1.0–8.6)	χ^2^ 8.9; OR 5.2 (1.6–18)	ns	χ^2^ 23.4; OR 0.78 (0.69–0.88)	ns	ns	ns
A	χ^2^ 8.1; OR 2.9 (1.4–6.1)	ns	ns	ns	ns	—	—	—	—
B2	ns	ns	χ^2^ 8.0; OR 3.7 (1.4–9.7)	ns	ns	—	—	—	—
D	χ^2^ 8.2; OR 0.19 (0.1–0.7)	χ^2^ 4.0; OR 3.0 (1.0–9.4)	ns	ns	ns	—	—	—	—

χ^2^ is Chi-square value, OR is odds ratio with 95% confidence interval, ns is not significant.

^1^The markers *stx1, stx2, eae. aggR, papA, safS, focG, ipaH, eltB, astA,* and phylogenetic group B1 showed no significant associations with any of the ESBL-genotypes or phylogenetic groups and were therefore omitted from this table.

**Table 2:** Prevalence of pathogenic variants among ESBL-producing *E. coli*.

Pathogenic category	Genetic marker	Surface water* (n = 82)	Waste water* (n = 88)	Total* (n = 170)	Phylogenetic group/ESBL-genotype
EAEC	*aggR*	5 (6.1%)	2 (2.2%)	7 (4.1%)	A_0_/CTX-M-15 (1)
					A_1_/CTX-M-15 (2)
					A_1_/CTX-M-27 (1)
					D_1_/CTX-M-15 (1)
					D_1_/CTX-M-14 (1)
					D_2_/CTX-M-15 (1)
EIEC	*ipaH*	0 (0%)	2 (2.3%)	2 (1.2%)	B2_3_/CTX-M-15 (1)
					D_2_/CTX-M-15 (1)
EPEC	*eae*	0 (0%)	3 (3.3%)	3 (1.8%)	B2_3_/CTX-M-15 (1)
					B1/CTX-M-15 (2)
ETEC	*eltB* or *estA*	2 (2.4%)	0 (0%)	2 (1.2%)	A_0_/CTX-M-15 (2)
ExPEC	≥3 markers	5 (6.1%)	10 (11.4%)	15 (8.8%)	B2_3_/CTX-M-1 (3)
					B2_3_/CTX-M-14 (1)
					D_2_/CTX-M-14 (2)
					A0/CTX-M-15 (1)
					B2_3_/CTX-M-15 (6)
					D_2_/CTX-M-15 (2)
STEC	*stx1* or *stx2*	0 (0%)	0 (0%)	0 (0%)	—
Total		12 (14.6%)	17 (19.3%)	29 (17.1%)	

^*^Indicated are the numbers and percentages of suspected pathogenic isolates, based on the presence of the indicated markers.

**Table 3:** Antibiotic resistance profiles of suspected pathogenic variants.

Pathogeniccategory	Phylogenetic group/ESBL-genotype	Sample ID^a^	Origin	ABR profile^b^	No. resistant classes
EAEC	A0/CTX-M-15	Sw5	Surface water	AmCxCzTm	2
	A1/CTX-M-15	Sw6	Surface water	AmCxCzTeNaStSuTm	5
	A1/CTX-M-27	Tp5	Airport WWTP	AmCxCzTeNaGeStSuTmCh	6
EIEC	B2_3_/CTX-M-15	Tp3	mWWTP	AmCxCzTeNaGeStSuTm	5
	D2/CTX-M-15	Tp5	Airport WWTP	AmCxCzTeCiNaGeSuTmCh	6
	A1/CTX-M-27	Tp5	Airport WWTP	AmCxCzTeNaGeStSuTmCh	6
EPEC	B1/CTX-M-15	Tp5	Airport WWTP	AmCxCzTeNaStSu	5
	B1/CTX-M-15	Tp3	mWWTP	AmCxCzNaTm	3
	B2_3_/CTX-M-15	Tp1.c	mWWTP	AmCxCzTeGeSuTm	4
ETEC	A0/CTX-M-15	Sw4.a	Surface water	AmCxCzTeNaSuTm	4
	A0/CTX-M-15	Sw4.b	Surface water	AmCxCzTeNaSuTm	4
ExPEC	A0/CTX-M-15	Sw1	Surface water	AmCxCzAxTe	3
	B2_3_/CTX-M-1	Sw1	Surface water	AmCx	1
	B2_3_/CTX-M-1	Tp1.a	mWWTP	AmCxTeCiNaStSuTm	5
	B2_3_/CTX-M-1	Tp2	mWWTP	AmCxAxTeNaGeStSuTm	6
	B2_3_/CTX-M-14	Sw2	Surface water	AmCxTe	2
	B2_3_/CTX-M-14	Sw1	Surface water	AmCxCzCiNaGe	3
	B2_3_/CTX-M-15	Sw3	Surface water	AmCxCzTe	2
	B2_3_/CTX-M-15	Hc1	HCI	AmCxCzAxTeCiNaGeStSuTm	6
	B2_3_/CTX-M-15	Hc2	HCI	AmCxCzAxTeCiNaStSuTm	6
	B2_3_/CTX-M-15	Tp1.b	mWWTP	AmCxCzTeNaGeStSuTm	5
	B2_3_/CTX-M-15	Tp1.c	mWWTP	AmCxCiNaSuTm	3
	B2_3_/CTX-M-15	Hc2	HCI	AmCxTeStSuTm	4
	D2/CTX-M-14	Tp3	mWWTP	AmCxNaGe	3
	D2/CTX-M-14	Hc2	HCI	AmCxCzTeNaGe	4
	D2/CTX-M-15	Tp4	mWWTP	AmCxCzTeCiNaTm	4

^a^Numbers are used to indicate different locations, the appending letters (a, b, or c) indicate different time-points; Sw = surface water, Tp = wastewater from WWTP, Hc = wastewater from health care institution.

^b^Am = ampicillin, Cx = cefotaxime, Cz = ceftazidime, Ax = amoxicillin + clavulanic acid, Te = tetracycline, Ci = ciprofloxacin, Na = nalidixic acid, Ge = gentamycin, St = streptomycin, Su = sulfisoxazol, Tm = trimethoprim, Ch = chloramphenicol, resistance was defined as disk diameters above clinical break-point (CLSI).
