# Supplementary material for: Pathogenic Escherichia coli producing Extended-Spectrum β-Lactamases isolated from surface water and wastewater
Source: Sci Rep. 2015 Sep 24;5:14372. doi: 10.1038/srep14372 (PMC4585870; doi:10.1038/srep14372)
Supplement: Supplementary info Table S1 [file srep14372-s1.pdf]

Supplementary Table S1.

Pathogenic *Escherichia coli* producing Extended-Spectrum β-Lactamases isolated from surface water and wastewater

Eelco Franz, Christiaan Veenman, Angela H.A.M. van Hoek, Ana de Roda Husman, and Hetty Blaak

Table S1. Primers and PCR conditions.

PCR specifics	PCR format[a]	Target	Primer name	Primer sequence (5'-3')	Amplicon size (bp)	Conc. (µM)	PCR programme	Reference
Phylogenetic group	Conventional, multiplex	*chuA*	ChuA.1	gacgaaccaacggtcaggat	279	0.2?	15min 95°C; 35x (15s 95°C, 20s 60°C, 30s 72°C); 5min 72°C	[1]
			ChuA.2	tgccgccagtaccaaagaca		0.2?		
		yjaA	YjaA.1	tgaagtgtcaggagacgctg	211	0.2?		
			YjaA.2	atggagaatgcgttcctcaac		0.2?		
		TspE4.C2	TspE4C2 66F	gagtaatgtcggggcattca	152	0.2?		
			TspE4C2 216R	cgcgccaacaaagtattacg		0.2?		
ESBL genotype	Conventional, multiplex	CTX-M-1 group	MultiCTXMGp1_for	ttaggaartgtgccgctgya	688	0.4	10min 95°C; 30x (40s 95°C, 40s 60°C, 60s 72°C); 7min 72°C	[2]
			MultiCTXMGp1-2_rev	cgatatcgttggtggtrccat		0.2		
		CTX-M-2 group	MultiCTXMGp2_for	cgttaacggcacgatgac	404	0.2		
			MultiCTXMGp1-2_rev	cgatatcgttggtggtrccat		0.2		
		CTX-M-9 group	MultiCTXMGp9_for	tcaagcctgccgatctggt	561	0.4		
			MultiCTXMGp9_rev	tgattctcgccgctgaag		0.4		
	Conventional, multiplex	TEM family	MultiTSO-T_for	catttccgtgtcgcccttattc	800	0.4	10min 95°C; 30x (40s 95°C, 40s 60°C, 60s 72°C); 7min 72°C	
			MultiTSO-T_rev	cgttcatccatagttgcctgac		0.4		
		SHV family	MultiTSO-S_for	agccgcttgagcaaattaaac	713	0.4		
			MultiTSO-S_rev	atcccgcagataaatcaccac		0.4		
		OXA family	MultiTSO-O_for	ggcaccagattcaactttcaag	564	0.4		
			MultiTSO-O_rev	gaccccaagtttcctgtaagtg		0.4		
EAEC	Conventional, singleplex	*aggR*	aggR 217F	gcaatcagattaaRcagcgataca	425	0.2	15min 95°C; 35x (30s 95°C, 30s 65°C, 30s 72°C); 10min	[3]
			aggR 642R	cattcttgattgcataaggatctgg		0.2		

							72°C	
EHEC	Real-time	*stx1*	stx1/2 F CRL	tttgtYactgtSacagcWgaagcYttacg	131	0.2	10min 95°C; 40x (15s 95°C, 30s 60°C)	[4]
			stx1/2 R CRL	ccccagttcaRWgtRagRtcMacRtc		0.2		
			stx1 probe CRL	FAM-ctggatgatctcagtgggcgttcttatgtaa-TAMRA		0.2		
	Real-time	*stx2*	stx1/2 F CRL	tttgtYactgtSacagcWgaagcYttacg	128	0.2	10min 95°C; 40x (15s 95°C, 30s 60°C)	
			stx1/2 R CRL	ccccagttcaRWgtRagRtcMacRtc		0.2		
			stx2 probe CRL	tcgtcaggcactgtctgaaactgctcc		0.2		
EHEC/EPEC	Real-time	*eae*	eae F CRL	cattgatcaggatttttctggtgata		0.2	10min 95°C; 40x (15s 95°C, 30s 60°C)	[5]
			eae R CRL	ctcatgcggaaatagccgtta		0.2		
			eae probe CRL	FAM-atagtctcgccagtattcgccaccaatacc-TAMRA		0.2		
EIEC	Conventional, singleplex	*ipaH*	ipaH_958F	ttccttgaccgcctttccg	611	0.2	10min 95°C; 35x (30s 95°C, 30s 60°C, 30s 72°C); 10min 72°C	[6]
			ipaH_1569F	agccaccctctgagagtac		0.2		
ETEC	Conventional, singleplex	*eltB*	eltB_32F	cggcgttactatcctctc	336	0.2	10min 95°C; 35x (30s 95°C, 30s 56°C, 30s 72°C); 10min 72°C	This study
			eltB_367R	ccatactgattgccgcaat		0.2		[7]
	Conventional, singleplex	*estA*	estA_47F	cacctttcgctcaggatgc	164	0.2	10min 95°C; 35x (30s 95°C, 30s 60°C, 30s 72°C); 10min 72°C	This study
			estA_210R	cccggtacaagcaggattac		0.2		
ExPEC	Conventional, multiplex	*focG*	focG_106F	cgtacctgtaccattggtaatggagg	366	0.2	15min 95°C; 35x (30s 95°C, 30s 60°C, 30s 72°C); 10min 72°C	[8] This study [9]
			R focG_471R	tgaattaatacttcccgcaccagc		0.2		
		kpsM II	kpsMII_121F	gcgcatttgctgatactgttg	452	0.2		
			kpsMII_572	gggaacatgatgcaggagatg		0.2		
		papA	papA_67F	atggcagtggtgtcttttggtg	717	0.2		
			papA_+202R	cgtcccaccatacgtgctcttc		0.2		
		sfaS	sfaS_210F	gtctctcaccggatgccagaatat	138	0.2		
			sfaS_347R	gcattacttccatccctgtcctg		0.2		

	Conventional, multiplex	*afa*	afa F	ggcagagggccggcaacaggc	594	0.2	15min 95°C; 35x (30s 95°C, 30s 60°C, 30s 72°C); 10min 72°C	
			afa R	cccgtaacgcgccagcatctc		0.2		
		hlyD	hlyD_92F	ctccggtacgtgaaaaggac	904	0.2		
			hlyD_995R	gccctgattactgaagcctg		0.2		
		iutA	iutA_674F	atcggctggacatcatgggaac	314	0.2		
			iutA_987R	cgcatttaccgtcgggaacgg		0.2		

[a]Used PCR formats: multiplex: Qiagen multiplex PCR mix (Qiagen Benelux B.V., Venlo, the Netherlands), singleplex and real-time: iQ-supermix (Bio-Rad Laboratories B.V., Veenendaal, the Netherlands);
EAEC= enteroaggregative *E. coli*, EHEC=enterohaemorrhagic *E. coli*, EIEC=enteroinvasive *E. coli*, EPEC=enteropathogenic *E. coli*, ETEC= enterotoxigenic *E. coli*, ExPEC=extraintestinal pathogenic *E. coli*.

1 Clermont, O., Bonacorsi, S. & Bingen, E. Rapid and simple determination of the *Escherichia coli* phylogenetic group. *Applied and Environmental Microbiology* **66**, 4555-4558 (2000).

2 Dallenne, C., da Costa, A., Decré, D., Favier, C. & Arlet, G. Development of a set of multiplex PCR assays for the detection of genes encoding important β-lactamases in Enterobacteriaceae. *Journal of Antimicrobial Chemotherapy* **65**, 490-495, doi:10.1093/jac/dkp498 (2010).

3 Boisen, N. *et al.* Genomic characterization of enteroaggregative *Escherichia coli* from children in Mali. *Journal of Infectious Diseases* **205**, 431-444, doi:10.1093/infdis/jir757 (2012).

4 Perelle, S., Dilasser, F., Grout, J. & Fach, P. Detection by 5′-nuclease PCR of Shiga-toxin producing *Escherichia coli* O26, O55, O91, O103, O111, O113, O145 and O157:H7, associated with the world's most frequent clinical cases. *Molecular and Cellular Probes* **18**, 185-192 (2004).

5 Nielsen, E. M. & Andersen, M. T. Detection and characterization of verocytotoxin-producing *Escherichia coli* by automated 5′ nuclease PCR assay. *Journal of Clinical Microbiology* **41**, 2884-2893 (2003).

6 Sethabutr, O. *et al.* Detection of shigellae and enteroinvasive *Escherichia coli* by amplification of the invasion plasmid antigen H DNA sequence in patients with dysentery. *Journal of Infectious Diseases* **167**, 458-461 (1993).

7 Tamanai-Shacoori, Z., Jolivet-Gougeon, A., Pommepuy, M., Cormier, M. & Colwell, R. R. Detection of enterotoxigenic *Escherichia coli* in water by polymerase chain reaction amplification and hybridization. *Canadian Journal of Microbiology* **40**, 243-249 (1994).

8 Jakobsen, L. *et al.* Broiler chickens, broiler chicken meat, pigs and pork as sources of ExPEC related virulence genes and resistance in *Escherichia coli* isolates from community-dwelling humans and UTI patients. *International Journal of Food Microbiology* **142**, 264-272, doi:10.1016/j.ijfoodmicro.2010.06.025 (2010).

9 Johnson, J. R. & Stell, A. L. Extended virulence genotypes of *Escherichia coli* strains from patients with urosepsis in relation to phylogeny and host compromise. *Journal of Infectious Diseases* **181**, 261-272, doi:10.1086/315217 (2000).
